# Empyema of the Antrum of Highmore; Its Relations to Other Diseases and Its Treatment

**Published:** 1909-12

**Authors:** 


### Empyema of the Antrum of Highmore; Its Relations to Other Diseases and Its Treatment

Wolff Freudenthal, of New York, gives the etiological factors in the causation of antral disease as dental caries, infection from extraction of teeth, nasal diseases, and tuberculosis. Epilepsy and dulness of intellect may result from chronic antrum disease, and stomach and intestinal symptoms due to swallowing of pus are sometimes seen. The diagnosis is aided by trans-illumination, oozing of pus through the nasal orifice of the antrum, and by *x*-ray pictures of the antrum. The disease may be treated conservatively in some cases with good results. In most cases some form of operation is demanded. Conservative treatment will consist of irrigation of the antrum. The antrum may be opened from the canine fossa, from the nose with removal of more or less of the bony tissue, and by a radical operation through the anterior wall of the antrum. It is necessary to remove all the diseased structures, but all undiseased bone and mucous membrane should be left intact.—*Medical Record,* October 23, 1909.

